# How Safe and Technical Is Modified Dunn Osteotomy in the Management of Patients with SCFE: A Clinical Trial with Short-Term Follow-Up

**DOI:** 10.1155/2023/2742083

**Published:** 2023-12-07

**Authors:** Amro Elsiofy, Mahmoud Elsherif, Moawed F. Eladawy, Tarek Mahmoud, Ahmed F. Sakr

**Affiliations:** Orthopedics, Suez Canal University, Ismailia, Egypt

## Abstract

**Objective:**

Over the last decade, modified Dunn osteotomy has been widely used in the management of slipped capital femoral epiphysis (SCFE) with varying degrees of complications. Different conclusions have been adopted. Our study represented our experience in using such a technique in stable and unstable SCFE and tried to determine its safety and applicability for routine practice.

**Methods:**

Our study adopted an interventional prospective design performed on 24 hips divided evenly between both sexes with a mean age of 13.25. On the Southwick classification, the cases were distributed between moderate and severe, which constituted 41.7% and 58.33%, respectively. Three quarters of the study subjects were stable according to the Loder classification. Each underwent modified Dunn osteotomy after a safe surgical hip dislocation.

**Results:**

Over the period of about 1-year follow-up, clinical evaluation was performed by examining the surgical site and assessing the legs' length, range of hip movement, Harris hip score, and iHOT-12 score. Radiological assessment was performed by calculation of slip angle from the frog lateral view, assessment of union, and occurrence of any complications. The study showed that there was significant improvement in patients in terms of radiological and clinical outcomes, with the occurrence of AVN in 16.7% of cases (4 out of 24). All cases of AVN occurred in unstable hips.

**Conclusion:**

Despite the complication of AVN, we believe the results of this study add to the current literature which suggests that modified Dunn osteotomy is an effective and safe technique for the management of moderate and severe SCFE. This trial is registered with PACTR202312819351504.

## 1. Introduction

Although pinning in situ is still the standard and preferred option for the majority of surgeons worldwide in the management of all stages of SCFE, recent trends towards finding the optimum technique and osteotomy to restore anatomical alignment and prevention of deformity have been widely utilized [1, 2].

Several proximal femoral osteotomies have been described at the subcapital, basicervical, intertrochanteric, and subtrochanteric levels. The percentage of osteonecrosis reported reached 10% to 100% following various operative approaches, and a combination of osteonecrosis and chondrolysis was reported in up to 42% of the patients [3–7].

One of these various techniques was Dunn osteotomy which comprised a subcapital osteotomy, thus aiming at anatomical reduction of the femoral head which is expected to function best biomechanically and to give the best prognosis. Unfortunately, it has been associated with a high rate of osteonecrosis of the femoral head, due to the blood supply's vulnerability to the epiphysis. Safe surgical hip dislocation, originally described by Ganz [8], preserves the physeal blood supply and allows subcapital osteotomy and complete removal of the posterior callus after peeling off the retinacular flap, complete correction of the slip angle, and restoration of the normal anatomy (modified Dunn osteotomy) [9, 10].

This study's rationale was to share our experience in the evaluation of the functional and radiological outcomes of modified Dunn osteotomy in the management of adolescents with moderate and severe SCFE. This included stable and unstable cases.

## 2. Patients and Methods

A prospective interventional design through an uncontrolled clinical trial was adopted to fulfill the purpose of the study from the period of February 2017 to June 2021. The study was conducted in the orthopedic surgery department at Suez Canal University Hospital in Ismailia. The included study population was all adolescents with moderate and severe SCFE presented to our hospital, including both stable and unstable cases. We excluded children who have reached skeletal maturity (closed physes), underwent previous osteotomy to correct the deformity, were unfit for surgery, or refused participation (Table 1).

A written informed consent was taken from the legal guardians of all participants and assert of the children before taking any data or performing any sort of investigation. Approval was obtained from the Research Ethical Committee of the faculty of medicine, Suez Canal University.

All participants were evaluated preoperatively through clinical and radiological assessment. Full medical history was taken from the study subjects and their parents including patient age, site of pain, onset of symptoms and its progress, any previous medications, previous surgery, or associated diseases. Physical examination was documented including BMI, range of hip movement, Harris hip score [11], and iHOT-12 score [12] (Table 2).

Radiological assessment was performed primarily through X-rays in both AP and frog lateral views and by calculation of slip angle. MRI of the pelvis and both hips was not performed regularly due to lack of resources.

Vascularity of the femoral head was just assessed intraoperatively through the drilling of the femoral head and no MRI perfusion protocol was initiated.

### 2.1. Surgical Technique

**Figure 1:**
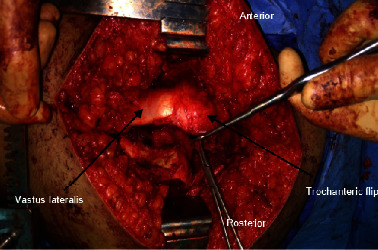
Trochanteric osteotomy is performed.

**Figure 2:**
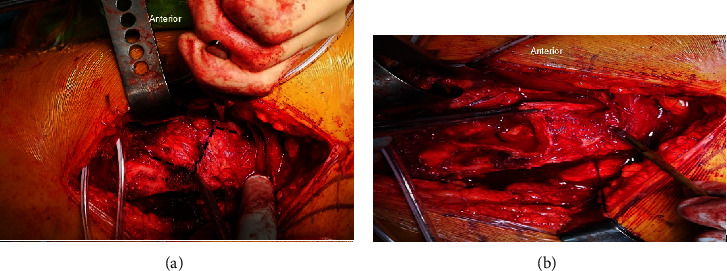
(a) The capsule is identified and *Z*-shaped capsulotomy site is marked. (b) Capsulotomy is performed in a *Z*-shaped pattern.

A 10 cm straight lateral incision centred over the greater trochanter was performed followed by incising the fascia lata. By leaving about 15 mm of posterior fibers of gluteus medius behind and freeing the vastus lateralis from bone, a straight digastric trochanteric osteotomy of about 1 cm depth was performed to flip the trochanteric fragment with the gluteus medius and vastus lateralis anteriorly (Figure 1). Underneath were the gluteus minimus fibers which were lifted off, thus exposing the capsule. A *Z*-shaped capsulotomy was then performed starting with the long arm running along the length of the femoral neck followed by a horizontal limb along the physis then finally curving anteriorly towards the lesser trochanter (Figure 2). All patients had their femoral epiphysis temporarily pinned with 2 K-wires. A drill hole was made in the epiphysis to check for prereduction epiphyseal perfusion and documented. Dislocating the femoral head after cutting the ligamentum teres was performed to allow full inspection of the corresponding damage to the acetabulum.

**Figure 3:**
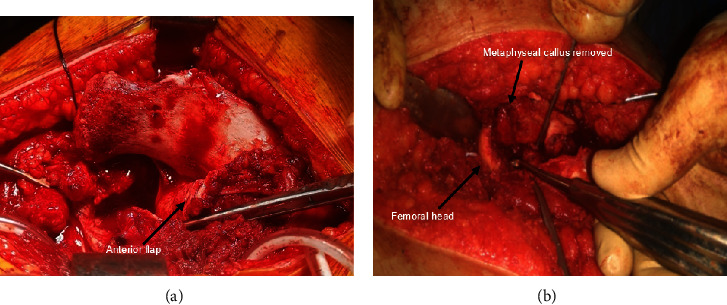
(a) The periosteum with the retinacular flap is incised carefully along the femoral neck. (b) Metaphyseal callus is removed and the shortening of the femoral neck is performed.

After relocation of the femoral head, the periosteum of the femoral neck was incised along its axis anteriorly and the retinacular soft tissue flap was then carefully mobilized with a sharp periosteal elevator. Distally and anteriorly, the flap was extended to the level of the lesser trochanter and then posteriorly the retinacular flap was mobilized fully after release from the posterior neck and extended distally to the greater trochanter. At that point, another trochanteric osteotomy through the posterosuperior portion of the apophysis including the piriformis fossa was performed and raised with the posterior flap down to the proximal femur. This extended release was intended to reduce the tension from the retinacular vessels. The femoral head was then mobilized using a chisel and a hammer starting anteriorly and then carefully a complete separation was achieved aided by gradual external rotation. Resection of the metaphyseal callus especially posteriorly was performed with further shortening of the neck performed in some patients who still had tense posterior retinacular flap upon reduction to avoid injury to the blood supply. Curettage of the epiphyseal growth plate was performed (Figure 3).

Reduction of the metaphysis over the epiphysis was performed under fluoroscopic guidance with abduction to avoid varus and then fixed by a 2 mm K-wire guided by ACL jig target guide for optimum position. Epiphyseal perfusion after capital realignment was checked using a 2 mm drill hole with observation of subsequent bleeding (video 1). Further fixation using 6.5 mm cancellous screws was performed under visualization with intraoperative imaging. The anterior periosteum and the posterior flap followed by the capsule were then reattached loosely. The greater trochanteric osteotomy was finally reduced and fixed using two 4.5 mm cortical screws followed by skin closure in layers.

### 2.2. Postoperative Management

Patients were allowed to leave the hospital when stable and safe on average 4 days postoperatively. Partial weight-bearing was allowed for eight weeks with crutches. Passive hip movement was encouraged immediately after surgery, and then after three weeks, all active motion except abduction was allowed. This was followed by a stepwise transition to full weight-bearing and normal use of the hip after three months.

**Figure 4:**
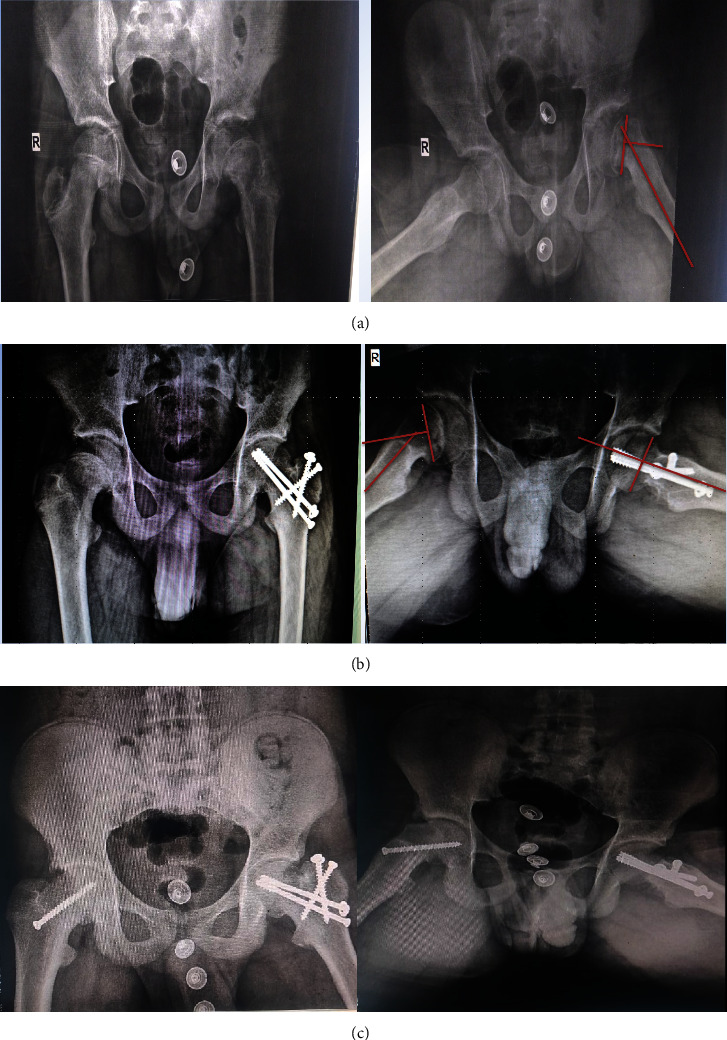
AP and frog lateral radiographs of a 14-year-old male patient with left stable chronic moderate SCFE. (a) Preoperative. (b) 1 year postoperative showing anatomical reduction and complete union of osteotomies and incidental finding of contralateral mild SCFE. (c) Pinning in situ of the right side and development of FAI which is listed for arthroscopic debridement.

All patients had follow-ups at two and six weeks and then at three-month, six-month, and one-year intervals (Figure 4).

Clinical evaluation was performed by examining the surgical site and assessing the legs' length, range of hip movement, Harris hip score, and iHOT-12 score. Radiologically, we measured the slip angle, assessed the union of the osteotomy sites, and checked for complications as AVN of the head femur.

Participants and their legal guardians were informed to seek advice whenever there was any discharge from the wound, severe pain, no response to medication, or acute limitation of movement.

### 2.3. Statistical Analysis

Collected data were coded, entered, and analysed using Microsoft® Office® Excel software. Data were then imported into SPSS version 20 for analysis. Baseline characteristics of the study population were presented as frequencies and percentages in qualitative data or as mean values and standard deviations in quantitative data.

Differences between frequencies were compared by chi-square or Fisher exact tests. Differences between means were compared by the *t*-test. A *P* value of less than 0.05 was considered significant. The Pearson correlation coefficient test was used to evaluate the intercorrelations between the studied variables. The analysis of variance (ANOVA) test followed by the logistic regression analysis model of the dependent variable and other standard variables (independent predictors) was performed where needed.

## 3. Results

A total of 24 patients were recruited in our study, and the male-to-female ratio was 1 : 1. The mean age was 13.25 ranging from 12 to 16 years with a mean body mass index of 28.78. The mean symptom duration was 1.88 months (±2 SD) with a range of 0.25–3. One patient had a previous surgery on the affected hip (pinning in situ) and none had any associated diseases.

Three quarters of the study subjects showed stable deformity according to Loder classification [13], while 42% of the hips were moderate and 58% severe as per Southwick classification [14].

The mean follow-up was 13 months (12 to 15). The median and mean hospital stay were around 4 days (range 2 to 11), while the median and mean surgery duration were around 2 hours (range 1.5 to 3.5). The mean intraoperative blood loss was 135 ml and there was no need for blood transfusion to any patient. There was a downtrend in the surgical time required from 3.5 hours in the first case to 1.5 hours in the last one. Four patients did not have any bleeding from their femoral head upon drilling before and after the reduction of the slipped head.

All wounds were dry on checking at 2 weeks postoperatively. At 6 weeks, all of the wounds showed full healing. There was a clear difference between the limb length discrepancy preoperatively and postoperatively with statistically significant results (*P* value of 0.011) where the mean discrepancy preoperative was 19 mm and the mean discrepancy postoperative was 2.75 mm.

**Figure 5:**
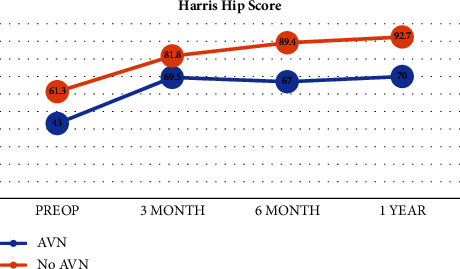
Mean Harris hip score measurements.

**Figure 6:**
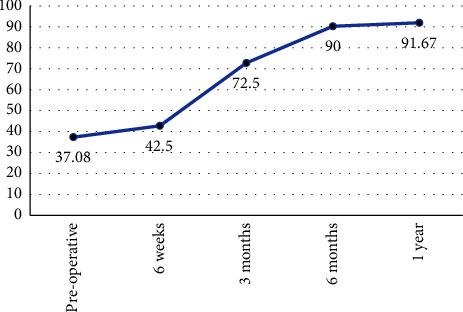
Mean iHOT-12 score measurements.

There was a remarkable boost in the hip range of movement after anatomical reduction of the femoral head (Table 3) as well as a marked improvement in the Harris hip score and iHOT-12 score (Figures 5 and 6).

Radiologically, there was significant improvement in the Southwick slip angle with a mean value of 64.17 (±16.12 SD) and a range of 37–85 preoperatively, while postoperatively, the mean Southwick slip angle was 1.67(±5.77 SD) with a range of 0–20.

The incidence of avascular necrosis of the femoral head was found in 4 patients discovered at 3 months postoperatively. There was a significant association between AVN and Loder classification with *P* value of <0.005 where all patients who suffered from AVN had unstable hips. Moreover, their femoral head did not bleed before and after relocation. With the development of head collapse, the implants were removed but no further operations were required.

No other complications including infection, chondrolysis, backing out of metalwork, or nonunion were experienced.

## 4. Discussion

The study shows that there was a significant improvement in the patients with moderate and severe SCFE after performing anatomical reduction by modified Dunn osteotomy in terms of radiological and clinical outcomes. More than half of our patients (58.3%) were classified as severe according to the Southwick classification and nearly half of those (42.8%) were classified as unstable as per the Loder classification. Eventually, even if the reduction was not perfect, patients with acute or chronic presentations still enjoyed a better quality of life after the operation than before.

The patients were allowed to stay in the hospital until safe enough to go home. That varied from two to eleven days with a mean hospital stay of 4.58. This variation was mainly due to different patients' responses and adherence to rehabilitation programs and postoperative instructions.

There was a clear learning curve in our study which was seen when analysing our data in terms of surgical time. At first, it started at 3.5 hours which was then improved in a linear downtrend to 1.5 hours at times with an average of 2.125 hours.

In our study, we measured the Southwick slip angle as a tool to assess the radiological outcome of performing the procedure. We operated on moderate and severe SCFE with a mean slip angle of 64.17 which has improved dramatically to a mean angle of 1.67 showing a significant difference. This was quite similar in other studies as the main aim of the procedure was an anatomical realignment of the femoral head on the neck as shown by Slongo et al. [5], Ziebarth et al. [9], and Zuo et al. [15] in their studies with slip angle reducing to a mean of 4.6°, 4.8°, and 7.5° at follow-up, respectively, as well as a mean correction of 43.42° and 43.63° in slip angle in studies performed by Abdelazeem et al. [6] and Agashe et al. [16], respectively. We can only assume that not all slips were reduced perfectly to zero degrees as in some severe cases, reducing the head anatomically did put more tension on the retinacular flap, thus a small slip was accepted as not to jeopardize the blood supply to the femoral head.

In terms of clinical outcome, we measured the hip range of motion in different directions including hip flexion, abduction, and internal and external rotations. The study showed noteworthy improvement in hip internal rotation with a mean correction of 36.6° after open reduction of the hip. Hip flexion and abduction did improve significantly as well from means of 55° flexion and 30° abduction preoperatively to 100° flexion and 52° abduction postoperatively. Similar studies also showed significant improvement in the hip range of movement as Abdelazeem et al. [6] with mean correction of flexion to 52° and internal rotation of 30° and another study by Hancıoğlu et al. [17] showed improvement of hip flexion from 85° to 105.6° and internal rotation from 0° to 28.3°. This marked improvement in hip range of motion was anticipated as we reduced the hip anatomically on the femoral neck. As a result of that reduction and bringing the neck from its external rotation, superior and anterior displacements and the degree of external rotation decreased to normal values postoperatively with a mean correction of −10°; however, this result was different from the study performed by Hancıoğlu et al. [17] which showed improved external rotation from 18.3° to 46.1°.

Another factor which has also improved after the anatomical reduction of the femoral head was the leg length discrepancy. The mean discrepancy preoperatively was 19 mm ranging from 10 to 30 mm. This has improved significantly postoperatively with a *P* value of 0.011 to a mean of just 2.75 mm ranging from 0 to 10 mm. Despite the anatomical reduction, few patients still developed limb length discrepancy and that was secondary to deliberate shortening of the femoral neck in some cases where the posterior retinacular flap was under tension despite removal of the posterior neck callus.

For collecting the clinical data and using a scoring system, we used the Harris hip score and iHOT-12 and compared them preoperatively and postoperatively. Our results showed a significant improvement from the mean Harris hip score of 58.25 preoperative to 67.42 just 6 weeks postoperative and further improvement to 79.75, 85.6, and 88.9 after 3, 6, and 12 months, respectively. More or less similar results were also achieved in other studies including Abdelazeem et al.'s [6] study where Harris hip score improved from a mean of 67.9 preoperative to 96.3 18 months postoperative. Also studies by Slongo et al. [5], Ziebarth et al. [9], Hueber et al. [18], Lerch et al. [10], Hancıoğlu et al. [17], and Zuo et al. [15] reported postoperative scores of 99, 99.6, 97.1, 94, 94.2, and 96.7, respectively. Our results show an improving Harris hip score with an upward trend but not reaching the 90s probably due to the shorter duration of follow-up as in comparison to other studies mentioned earlier. However, the results were similar to studies of Agashe et al. [16] who in spite of long-term follow-up also had a final HHS of 81.8. The other issue we encountered was the noncompliance of some patients to strictly adhere to their rehabilitation program. There was the inadequacy of physiotherapy, especially for patients with low socioeconomic status who live in rural areas in the Suez Canal region, who eventually needed longer rehab time and suboptimal clinical outcomes.

One of the complications we encountered was the occurrence of avascular necrosis of the femoral head which affected 16.7% of patients. These 4 patients did not show any bleeding from the femoral head upon drilling intraoperatively which was highly significant and all of them were classified as unstable hips. Two of these patients were a revision of a severe SCFE who had pinning in situ and developed chondrolysis. Eventually after 1 year postoperatively, these patients developed a collapse of the femoral head and only the removal of the implants was performed. They did improve upon clinical outcome but not dramatically with limitations in comparison to other patients as shown by the statistically significant association between AVN and both Harris Hip score and iHOT-12 (Figures 5 and 6).

Apart from a reported study by Zeibarth et al. [9] who operated on 40 patients showing no cases of AVN, our study when compared with other studies showed a percentage of AVN within average as Upasani et al. [19] in 2014 showed 23.2% from a total of 43 patients. Slongo et al. [5] in 2010 who reviewed 23 patients showed that 4.3% developed AVN. Madan et al. [20] in 2013 reported 14% of AVN in 28 patients. Also in 2013, Sankar et al. [21] reported 26% of AVN among 27 patients in total, and Abdelazeem et al. [6] in 2016 who operated on 31 patients reported 1 case of AVN (3.2%). More recent data included studies from Novais et al. [22] in 2018 who compared between modified Dunn osteotomy and pinning in situ and his results showed the occurrence of AVN in the first group to be 26%. Moreover, studies by Masquijo et al. [23] in 2019, Lerch et al. [10] in 2019, Ebert et al. [7] in 2019, Agashe et al. [16] in 2020, Hancıoğlu et al. [17] in 2020, and Zuo et al. [15] in 2020 reported 29%, 5%, 26%, 6.6%, 11.1%, and 0% of AVN, respectively. There is still some discrepancy in results where a recent study by Fournier et al. [24] published in 2022 who operated on unstable SCFE using modified Dunn osteotomy had AVN in 33% of cases and shifted their protocol of treatment after 2012 to undergo anterior cuneiform osteotomy instead; however, despite the occurrence of AVN in 8.7% in the latter group, yet analysis didnot find any relationship between AVN and the type of treatment.

**Figure 7:**
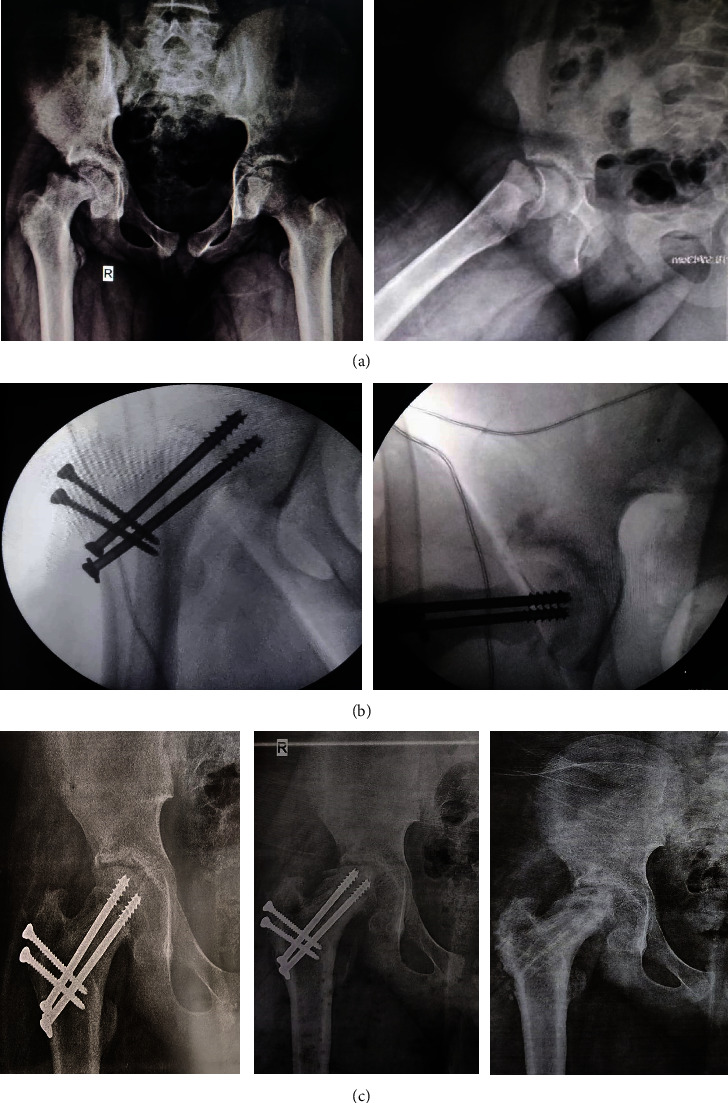
Radiographs of a 14-year-old male patient with right unstable severe SCFE. (a) Preoperative. (b) Intraoperative showing anatomical reduction. (c) Postoperative AP images at 3, 6, and 12 months follow-up showing signs of AVN starting at 3-month mark.

The incidence of AVN was clearly seen on X-ray just 3 months postoperatively (Figure 7). It was not clear or documented in the literature about the timing of the occurrence of AVN; however, we believed after one year of follow-up, no other patient would develop AVN.

There was a total of 4 patients who developed AVN in our study, and all were classified as unstable hips. These patients were unable to ambulate and were bedridden preoperatively, and although that changed postoperatively, they still did develop AVN. In analysing these data, it showed a statistically significant positive correlation between Loder classification and the occurrence of avascular necrosis of the femoral head. We assume that this is not related to the surgical technique but more due to the nature of instability of the femoral head in these patients which could result in kinking of the blood vessels supplying the epiphysis. This conclusion was quite similar to a recent study by Mallet et al. [25] who performed an anterior subcapital shortening osteotomy for the treatment of severe slipped SCFE and the incidence of AVN occurred in 16 out of 144 cases in total; 75% of those had an unstable hip. This conclusion however was different from a similar study performed by Rebello et al. [26] who found three of four cases of osteonecrosis occurring in patients who had undergone femoral neck or intertrochanteric osteotomy for deformities resulting from stable SCFE and he assumed that the result was due to technical difficulty found in resection of the physis and callus found in stable slippage. In addition to that, another study by Davis et al. [27] in 2019 reported to have a higher rate of AVN in stable SCFE and it was concluded that the complication rate was related to the complexity of the manoeuvre of reconstruction at the time of dislocation.

In our study, no regular preoperative or any postoperative MRI was performed on patients as we lacked resources and we believed it would not affect the outcome. We presumed that open reduction would at least restore the hip anatomy in even AVN susceptible patients and would be beneficial for later hip arthroplasty.

No other complications including infection, chondrolysis, backing out of metalwork, or nonunion were experienced.

The main strength of our study was the presence of one main surgical team to operate. All the patients were compliant with our follow-up appointments and there were no drop-outs in our study. It was clear and easy to measure the parameters of the clinical and radiological outcomes.

There were some limitations to our study confined to a small sample size although we included all eligible cases in over 4 years. Our study did have a short follow-up period which is not preferred although patients were back to their normal activities and it was adequate to identify complications including the occurrence of AVN. This was a clinical trial with no control group. Variables such as physiotherapy being performed at various sites with dissimilar techniques did affect our results in terms of clinical and functional outcomes. In our study, no routine MRI was performed on all patients as a preoperative tool to assess the viability of the femoral head.

## 5. Conclusion

The study would add to the literature after achieving its aim through the assessment of modified Dunn osteotomy after surgical hip dislocation in the management of moderate and severe SCFE showing promising results in achieving the optimal functional and radiological outcomes for the patients.

Although we came across AVN in 16.7% of cases, all occurring in unstable hips, we believe this complication is due to the primary irreversible vascular insult rather than any technical issue whilst operating.

## Figures and Tables

**Table 1:** Total number of children with moderate and severe SCFE.

Included		24

Excluded	Previous osteotomy	2
	Closure of physis	2
	Refusal of participation	4
	Unfit for surgery	0

**Table 2:** Mastersheet of the study population.

No.	Demographic and historic data				Investigations preoperation								Drilling of the femoral head	F/U at 3 months						F/U at 6 months			F/U at 1 year		
	Age	Sex	BMI	Site	HHS	iHOT-12	LLD (mm)	Internal rotation	External rotation	Southwick slip angle	SSA classification	Loder classification		X-ray	HHS	iHOT-12	LLD (mm)	Internal rotation	External rotation	X-ray	HHS	iHOT-12	X-ray	HHS	iHOT-12
1	13	Male	25.4	Right	66	55	5	10	40	37	Moderate	Stable	Bleeding		79	70	0	45	45		81	90		83	90
2	12	Female	26	Right	52	30	10	0	60	68	Severe	Stable	Bleeding		75	65	0	40	40		83	100		85	88
3	12	Female	35.7	Left	56	39	14	10	40	45	Moderate	Stable	Bleeding		79	75	0	45	45		89	100		92	95
4	15	Male	24.2	Left	80	69	21	0	60	50	Moderate	Stable	Bleeding		89	95	0	50	50		96	100		98	100
5	16	Male	34.7	Right	52	35	20	0	60	70	Severe	Stable	Bleeding		79	65	0	45	45		85	100		90	94
6	14	Female	24.1	Left	70	60	15	0	50	55	Moderate	Stable	Bleeding		83	70	0	45	45		95	100		100	100
7	13	Female	30.4	Right	51	20	25	0	60	70	Severe	Stable	Bleeding		81	80	0	30	50		86	90		93	93
8	13	Male	29.9	Right	56	55	20	0	40	50	Moderate	Stable	Bleeding		79	75	0	30	50		84	95		87	95
9	13	Male	29.4	Right	52	21	26	0	60	70	Severe	Stable	Bleeding		82	85	0	30	50		87	90		94	93
10	13	Female	30.9	Left	55	54	19	0	40	50	Moderate	Stable	Bleeding		78	75	0	30	50		83	95		86	95
11	12	Female	27	Right	53	36	21	0	60	72	Severe	Stable	Bleeding		80	70	0	45	45		86	100		91	94
12	13	Female	33.5	Right	71	61	26	0	70	80	Severe	Stable	Bleeding		85	80	0	45	45		97	100		98	100
13	14	Female	33.8	Left	44	9	24	0	75	85	Severe	Unstable	No bleeding	AVN	68	50	0	20	55	AVN	68	60	AVN	70	70
14	14	Male	33.5	Left	70	60	25	0	70	80	Severe	Stable	Bleeding		84	85	0	45	45		96	100		98	100
15	13	Female	25.2	Right	45	10	25	0	75	85	Severe	Unstable	No bleeding	AVN	69	55	0	20	55	AVN	69	60	AVN	70	70
16	13	Male	33.8	Left	79	70	20	0	60	50	Moderate	Stable	Bleeding		90	90	0	50	50		95	100		98	100
17	14	Male	26.6	Right	41	0	29	0	90	70	Severe	Unstable	No bleeding	AVN	71	55	0	20	60	AVN	65	45	AVN	70	75
18	14	Male	24	Left	67	56	6	10	40	35	Moderate	Stable	Bleeding		80	75	0	45	45		82	90		84	90
19	12	Male	22	Right	59	10	20	0	70	80	Severe	Unstable	Bleeding		88	75	0	45	45		98	100		100	100
20	12	Female	34.7	Both	57	40	15	10	40	45	Moderate	Stable	Bleeding		80	80	0	45	45		90	100		93	95
21	12	Male	24.4	Left	51	29	9	0	60	65	Severe	Stable	Bleeding		74	60	0	40	40		82	100		84	88
22	15	Male	32.7	Right	69	59	14	0	50	55	Moderate	Stable	Bleeding		82	65	0	45	45		94	100		100	100
23	14	Female	23.1	Left	60	11	21	0	70	80	Severe	Unstable	Bleeding		89	80	0	45	45		99	100		100	100
24	14	Female	25.6	Left	42	0	30	0	90	80	Severe	Unstable	No bleeding	AVN	70	60	0	20	60	AVN	66	45	AVN	70	75

**Table 3:** Hip range of movement preoperatively and postoperatively.

Hip range of movement	Preoperative range (mean)	Postoperative (3-month mark) range (mean)	*P* value
Flexion	10°–80° (55°)	60°–120° (100°)	0.025
Abduction	10°–40° (30°)	40°–60° (52°)	0.015
Adduction	10°–25° (20°)	10°–40° (26°)	0.064
Internal rotation	0°–10° (1.67°)	20°–45° (38.3°)	0.01
External rotation	40°–90° (60°)	40°–60° (50°)	0.06

## Data Availability

The data worksheet used to support the findings of this study and Video 1 which shows an intraoperative scene with bleeding femoral head have been deposited in the FAIRsharing (Zenodo) repository (DOI 10.5281/zenodo.7964134).
